# LncRNA *3222401L13Rik* Is Upregulated in Aging Astrocytes and Regulates Neuronal Support Function Through Interaction with Npas3

**DOI:** 10.3390/ncrna11010002

**Published:** 2025-01-09

**Authors:** Sophie Schröder, M. Sadman Sakib, Dennis M. Krüger, Tonatiuh Pena, Susanne Burkhardt, Anna-Lena Schütz, Farahnaz Sananbenesi, André Fischer

**Affiliations:** 1 Department for Systems Medicine and Epigenetics, German Center for Neurodegenerative Diseases (DZNE), 37075 Göttingen, Germany; sophie.schroeder@dzne.de (S.S.); m.sadman.sakib@gmail.com (M.S.S.); dennis.krueger@dzne.de (D.M.K.); tonatiuh.pena@dzne.de (T.P.); susanne.burkhardt@dzne.de (S.B.); 2 Bioinformatics Unit, German Center for Neurodegenerative Diseases (DZNE), 37075 Göttingen, Germany; 3 Research Group for Genome Dynamics in Brain Diseases, German Center for Neurodegenerative Diseases, 37075 Göttingen, Germany; anna-lena.schuetz@dzne.de (A.-L.S.); fsananb@gwdg.de (F.S.); 4 Cluster of Excellence “Multiscale Bioimaging: From Molecular Machines to Networks of Excitable Cells” (MBExC), University of Göttingen, 37075 Göttingen, Germany; 5 Department of Psychiatry and Psychotherapy, University Medical Center Göttingen, 37075 Göttingen, Germany

**Keywords:** lncRNA, brain, aging, Alzheimer’s disease, neurodegenerative diseases, astrocytes, non-coding RNA, transcriptomics

## Abstract

Aging leads to cognitive decline and increased risk of neurodegenerative diseases. While molecular changes in central nervous system (CNS) cells contribute to this decline, the mechanisms are not fully understood. Long non-coding RNAs (lncRNAs) are key regulators of cellular functions. **Background/Objectives:** The roles of lncRNAs in aging, especially in glial cells, are not well characterized. **Methods:** We investigated lncRNA expression in non-neuronal cells from aged mice and identified 3222401L13Rik, a previously unstudied lncRNA, as upregulated in astrocytes during aging. **Results:** Knockdown of 3222401L13Rik in primary astrocytes revealed its critical role in regulating genes for neuronal support and synapse organization, a function conserved in human iPSC-derived astrocytes. A 3222401L13Rik interacts with the transcription factor Neuronal PAS Domain Protein 3 (Npas3), and overexpression of Npas3 rescues deficits in astrocytes lacking 3222401L13Rik. **Conclusions:** These data suggest that 3222401L13Rik upregulation may help delay age-related cognitive decline.

## 1. Introduction

Aging is a major risk factor for the onset and progression of neurodegenerative diseases [1]. Even in the absence of pathological conditions, aging is associated with a decline in cognitive functions in both mice and humans [2,3,4] (Stern, 2012) [5]. Structural alterations in the aging brain include decreases in neuronal spine length and density, reduced memory-related neuronal firing, and changes in glial cell function, including altered astrocyte [6,7,8] and microglia function [9,10]. At the molecular level, shifts in gene expression have been described, reflecting processes such as synaptic transmission, vesicular transport, energy production, and immune activation [2,3,11,12]. However, the mechanisms underlying these changes in gene expression are still not fully understood, particularly concerning glial cells, when compared to neurons.

The non-coding RNAome has emerged as a critical regulator of gene expression and other cellular functions [13], offering novel and so far unexplored opportunities for therapeutic interventions [14]. Among non-coding RNAs, long non-coding RNAs (lncRNAs) represent a heterogeneous class of RNAs longer than 300 nucleotides that lack coding potential [13,15]. Key characteristics of lncRNAs, in comparison to protein-coding RNAs, include their relatively lower abundance, preferential nuclear localization, greater tissue- and cell-specific expression patterns, and comparatively lower sequence conservation across species [16]. Due to these features, lncRNAs were long considered transcriptional noise, with research and drug discovery focusing primarily on protein-coding transcripts [17,18]. However, recent research has highlighted the significant roles of lncRNAs in physiological and pathological conditions, impacting a wide array of biological processes, including genome architecture regulation, gene expression modulation, splicing regulation, and control of protein translation and localization [13]. Additionally, the observation that approximately 40% of human lncRNAs exhibit brain-specific expression patterns suggests that these molecules play complex roles in brain development and function [19], as well as in the pathogenesis of central nervous system disorders [18,20,21]. Several studies have demonstrated that lncRNAs are critical in cognitive diseases [22,23,24,25,26,27,28].

Given the limited research on the role of lncRNAs in the aging brain and the sparse data on non-neuronal glial cells, this study aimed to identify lncRNAs differentially expressed in the hippocampus of aged versus young mice and to investigate the functional implications of candidate lncRNAs. Our findings reveal that one such deregulated lncRNA, *3222401L13Rik*, which has not been previously studied, is specifically upregulated in astrocytes of aged mice. Antisense oligonucleotide-mediated knockdown of *3222401L13Rik* in primary astrocytes, followed by total RNA sequencing, demonstrates its crucial role in regulating genes essential for neuronal support functions. This regulation impacts astrocytic processes such as glutamate uptake and calcium signaling, leading to disruptions in neuronal network plasticity and synapse density. Additionally, we show that its human homolog, *ENSG00000272070*, regulates similar genes and functions in human iPSC-derived astrocytes. Furthermore, we demonstrate that *3222401L13Rik* exerts its effects by interacting with and regulating the transcription factor Neuronal PAS Domain Protein 3 (Npas3). Importantly, overexpression of *Npas3* is sufficient to rescue the functional deficits observed in astrocytes lacking *3222401L13Rik*.

## 2. Results

### 2.1. Total RNA Sequencing of Glia of Young and Aged Mice Identifies Differentially Regulated lncRNAs

**Figure 1:**
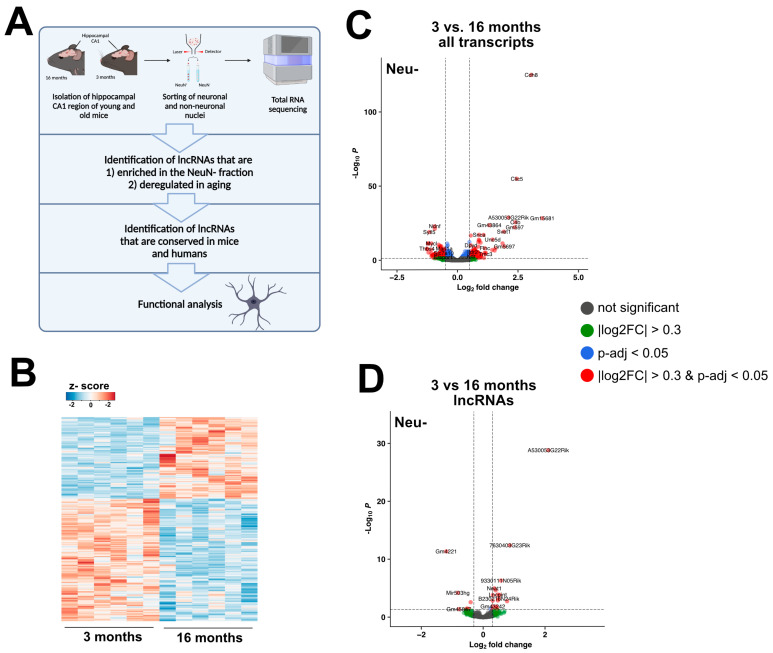
Aging induces changes in glial lncRNA expression patterns. (**A**) Schematic illustration of the experimental approach of this study. (**B**) Heatmap showing gene expression changes in Neu– nuclei isolated in 3- vs. 16-month-old mice. (**C**) Volcano plot showing the up- and downregulated coding transcripts when comparing Neu– nuclei from 3 vs. 16-month-old mice (log2fold changes are depicted as 16/3 months). (**D**) Volcano plot showing expression changes in lncRNAs in Neu– nuclei (log2fold changes are depicted as 16/3 months).

To identify deregulated lncRNAs in glia during aging, we isolated tissue from the hippocampal CA1 region of young (3 months) and old (16 months) mice. The hippocampus is a brain region crucial for memory function, and aging significantly affects this area. At 16 months, cognitive decline becomes observable in mice [3]. We processed the tissue to isolate nuclei via fluorescence-associated nuclei sorting (FANS), using neuronal nuclear protein (NeuN) to distinguish between neuronal (NeuN+) and non-neuronal nuclei (NeuN−) fractions. These two populations were then subjected to total RNA sequencing (Figure 1A).

We aimed to identify lncRNAs that are enriched in the NeuN− fraction and deregulated during aging (Figure 1A). To achieve this, we first analyzed differentially regulated genes in the NeuN− populations of young and aged mice (Figure 1B,C). In comparison to young mice, 187 genes were upregulated, and 310 genes were downregulated in 16-month-old mice (|log2FC| > 0.3, FDR < 0.05, base mean > 50) (Table S1). Filtering for lncRNAs from this list, we identified 17 upregulated and 7 downregulated lncRNAs (Figure 1D). Although our analysis focused on NeuN− nuclei, the 24 deregulated lncRNAs may also be expressed in NeuN+ nuclei. To determine if any of these lncRNAs were particularly enriched in the NeuN− population, we compared gene expression profiles of the NeuN+ and NeuN− fractions. Substantial differences were observed, with 51 lncRNAs highly enriched in NeuN− nuclei (Table S2).

Next, we compared the list of lncRNAs deregulated during aging (*n* = 24) with those enriched in the NeuN− fraction (n = 51). To facilitate the translation of our findings to human studies, we focused on lncRNAs with human homologs. Among the 24 deregulated lncRNAs in 16-month-old mice, seven were significantly enriched in the NeuN− fraction compared to NeuN+ nuclei (log2FC > 4, padj < 0.05), with only three having human homologs. These included nuclear enriched abundant transcript 1 (*Neat1*) and *3222401L13Rik*, both upregulated in 16-month-old mice, and *C430049B03Rik*, which was downregulated (Table 1). Since *Neat1* has been extensively studied, particularly concerning its role in astrocytes [25], and *C430049B03Rik* has been analyzed as a host gene for miR-322, miR-503, and miR-531 in mouse embryonic fibroblasts [29], we chose to focus our study on *3222401L13Rik*, a lncRNA that has not been previously characterized in any organ.

### 2.2. 3222401L13Rik Is a Glial-Enriched lncRNA Showing Increased Expression in Astrocytes of Aged Mice

**Figure 2:**
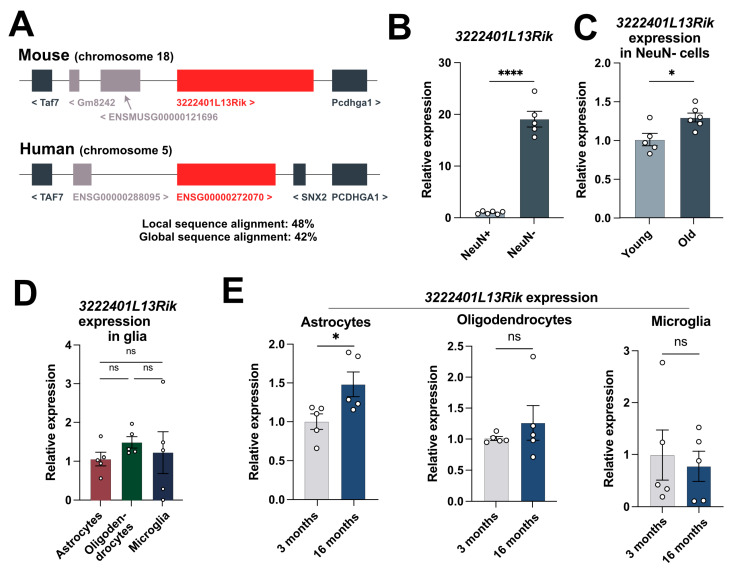
A *3222401L13Rik* is a glial lncRNA that is upregulated in astrocytes during aging. (**A**) Schematic illustration of the genomic localization of *3222401L13Rik* in the mouse and *ENSG00000272070* in the human genome. (**B**) Expression of the lncRNA *3222401L13Rik* in NeuN+ and NeuN− cells isolated from the hippocampal CA1 region of 3-month-old mice (unpaired *t*-test; **** *p* < 0.0001). (**C**) qPCR data showing the expression of *3222401L13Rik* in NeuN− cells from 3- and 16-month-old mice (unpaired *t*-test; * *p* < 0.05). (**D**) Expression of *3222401L13Rik* in astrocytes, oligodendrocytes, and microglia isolated from the brains of 3-month-old mice using MACS (One-way ANOVA; ns = not significant). (**E**) Expression of *3222401L13Rik* in astrocytes, oligodendrocytes, and microglia isolated from the brains of 3- and 16-month-old mice using MACS technology (unpaired *t*-test; * *p* < 0.05, ns = not significant). Data are depicted as mean ± standard error.

A *3222401L13Rik* is a long intergenic lncRNA (lincRNA) located on chromosome 18 in the mouse genome, with its human homolog (ENSG00000272070) found on chromosome 5 (Figure 2A). We confirmed the total RNA sequencing results with qPCR, demonstrating that 3222401L13Rik is predominantly expressed in NeuN− compared to NeuN+ nuclei (Figure 2B) and exhibits increased expression in 16-month-old mice (Figure 2C). Since the NeuN−fraction includes all brain cell types except neurons, we further investigated the expression pattern of *3222401L13Rik* in glial cells by performing Magnetic Activated Cell Sorting (MACS) of astrocytes, oligodendrocytes, and microglia from 3-month-old mice. qPCR analysis showed that *3222401L13Rik* expression was comparable across all three glial cell types analyzed (Figure 2D). However, when comparing expression levels in 3-month-old and 16-month-old mice, we observed an increase in *3222401L13Rik* levels only in astrocytes but not in oligodendrocytes or microglia (Figure 2E). Consequently, we focused on characterizing the role of 3222401L13Rik, specifically in astrocytes.

### 2.3. Loss of 3222401L13Rik Leads to Expression Changes in Genes Involved in Neuronal Support and Inflammatory Processes

**Figure 3:**
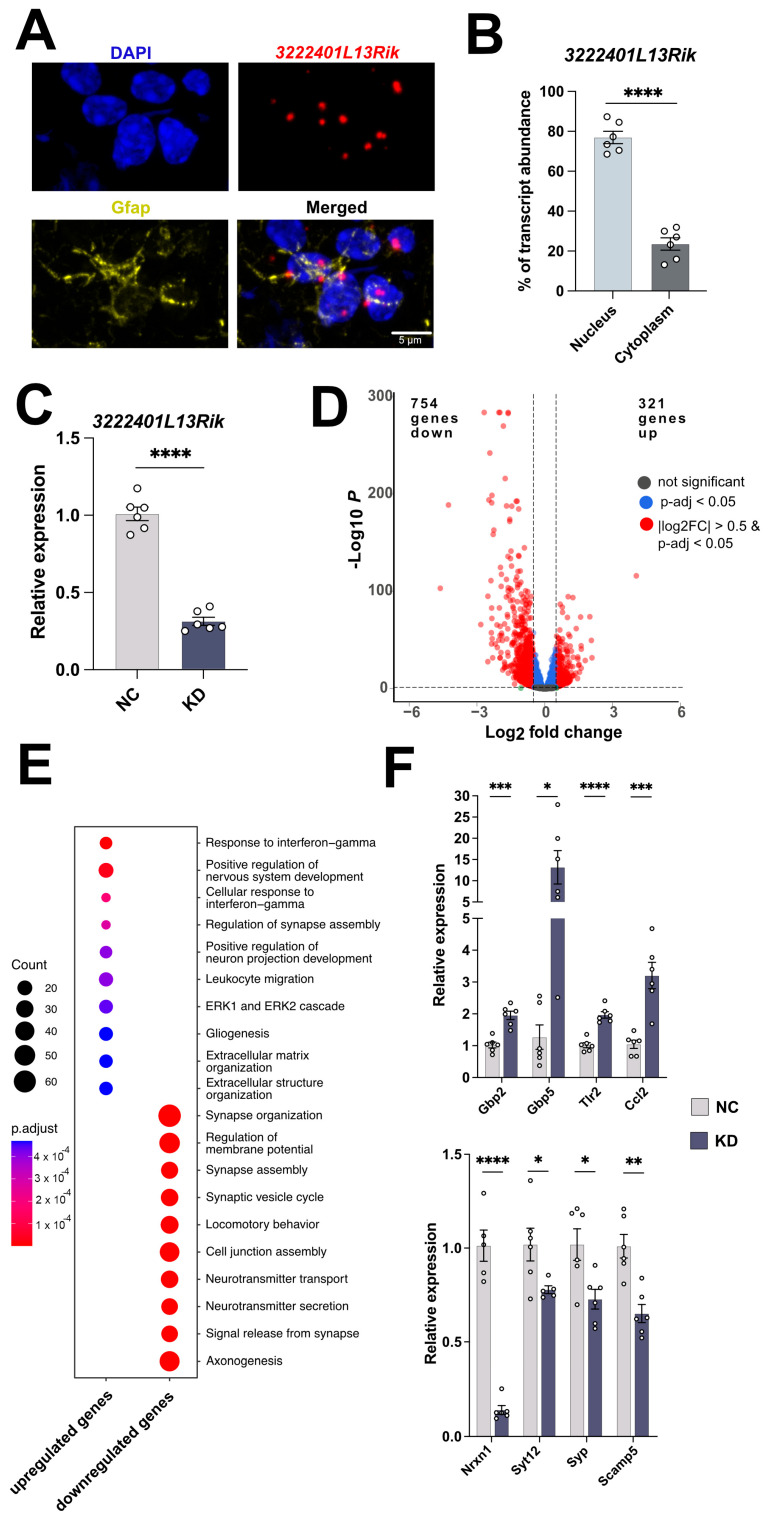
*3222401L13Rik* controls the expression of genes linked to innate immune response and synaptic support functions. (**A**) Representative image showing the nuclear localization of *3222401L13Rik* (RNAscope) in astrocytes (immunofluorescence for Gfap) in the adult mouse brain. Nuclei are stained using DAPI. (**B**) Bar chart showing the results of a qCPR that analyzes the expression of *3222401L13Rik* in nuclear and cytoplasmic fractions isolated from primary astrocytes (unpaired *t*-test; **** *p* < 0.0001). (**C**) Bar charts showing qPCR results to measure the expression levels of *3222401L13Rik* after treatment with NC or KD ASOs (**** *p* < 0.0001). (**D**) Volcano Plot showing the up- and downregulated genes 48 h after the KD of *3222401L13Rik* in primary astrocytes (log2fold changes are depicted as KD/WT). (**E**) Gene Ontology analysis of the genes shown in (**D**). Analysis was performed using clusterProfiler (v4.6.0) [31]. (Two-sided hypergeometric test was used to calculate the importance of each term, and the Benjamini–Hochberg procedure was applied for *p*-value correction). (**F**) Expression levels of selected genes that were deregulated after the KD of *3222401L13Rik*. Upper panel: upregulated genes. Lower panel: downregulated genes (unpaired *t*-test; * *p* < 0.05, ** *p* < 0.01, *** *p* < 0.001, **** *p* < 0.0001). Data are depicted as mean ± standard error. NC: negative control, KD: knockdown of *3222401L13Rik*.

lncRNAs can exert a wide range of functions, which primarily depend on their location within the cell, and the identification of their subcellular localization is, therefore, critical for their characterization [30]. Although we identified *3222401L13Rik* in Neu– nuclei, this does not exclude the possibility that it could be prominently expressed in the cytoplasm. Therefore, we investigated the localization of *3222401L13Rik* by combining RNAscope for *3222401L13Rik* with immunofluorescence staining for the astrocyte marker glial fibrillary acidic protein (Gfap) and found that *3222401L13Rik* was predominantly located in the nucleus of astrocytes in the adult mouse brain (Figure 3A). Moreover, strikingly, we observed two to three large foci of *3222401L13Rik* per astrocyte (Figure 3A). We confirmed the nuclear localization by performing nuclear and cytoplasmic fractionation of primary mouse astrocytes and measuring the abundance of *3222401L13Rik* in both compartments (Figure 3B). The enrichment of *3222401L13Rik* in the nuclear compartment hints toward the role of this lncRNA in regulating gene transcription. Therefore, we performed a knockdown (KD) of *3222401L13Rik* in primary mouse astrocytes using Gapmers directed against the lncRNA and performed total RNA sequencing to identify transcriptional targets. As a negative control (NC), a Gapmer with no known target in the genome was employed. Gapmers are single-stranded antisense oligonucleotides that mediate RNase H-dependent degradation of RNA, a technique that is especially suitable for targeting nuclear lncRNAs. We confirmed by qPCR that the Gapmers reduced the abundance of *3222401L13Rik* by ~75% (Figure 3C). Differential expression analysis after total RNA sequencing revealed that 321 genes were upregulated and 754 genes were downregulated upon *3222401L13Rik* KD (|log2FC| > 0.5, *p*-adj. < 0.05, base mean > 50) (Figure 3D) (Table S3). The upregulated genes were linked with a number of interesting Gene Ontology (GO) terms, including “Response to interferon-gamma”, “Positive regulation of nervous system development”, “Leukocyte migration”, and “Extracellular matrix organization” (Figure 3E) (Table S4). The downregulated genes were associated with GO terms such as “Synapse organization”, “Regulation of membrane potential”, “Synaptic vesicle cycle”, and “Locomotory behavior” (Figure 3E) (Table S4). To confirm the RNA sequencing data, we performed a qPCR for several of the upregulated genes that were part of the signaling pathway “Response to interferon-gamma” and downregulated genes linked to synaptic function (Figure 3F).

### 2.4. 3222401L13Rik Is Important for Glutamate Uptake, Ca^2+^ Signaling, and Neuronal Support

**Figure 4:**
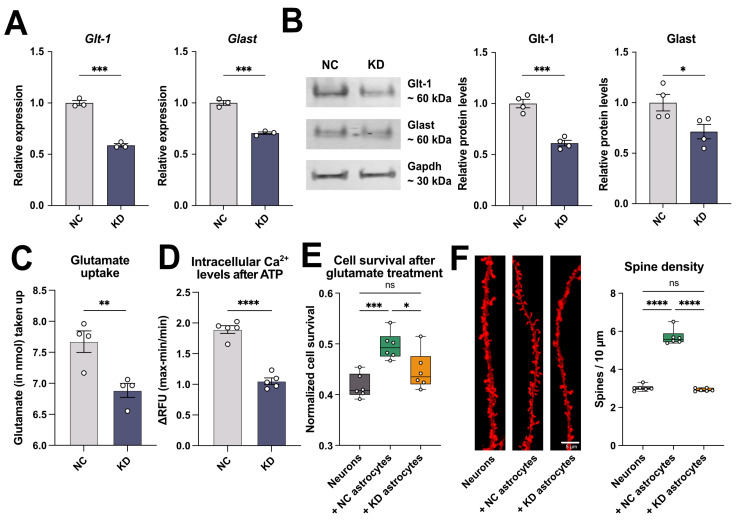
The KD of *3222401L13Rik* affects glutamate uptake, Ca^2+^ signaling, and the support of neuronal function. (**A**) qPCR showing the expression levels of the glutamate transporters *Glt-1* and *Glast* after the KD of *3222401L13Rik* in primary astrocytes (unpaired *t*-test; *** *p* < 0.001). (**B**) Left panel: Representative immunoblot images of Glt-1 and Glast following the KD of *3222401L13Rik*. in primary astrocytes. Right panel: Quantification of the left panel (unpaired *t*-test; * *p* < 0.05, *** *p* < 0.001). (**C**) Glutamate uptake of primary astrocytes after the KD of *3222401L13Rik* (unpaired *t*-test; ** *p* < 0.01). (**D**) Increase in intracellular Ca^2+^ levels in response to ATP treatment after the KD of *3222401L13Rik* (unpaired *t*-test; **** *p* < 0.0001). (**E**) Survival of neurons after treatment with 100 µM glutamate cultured alone or co-cultured with NC or KD astrocytes (One-way ANOVA; * *p* < 0.05, *** *p* < 0.001, ns = not significant). (**F**) Left panel: Representative images of dendrite and spine labeling of neurons cultured alone or co-cultured with NC or KD astrocytes. Right panel: Quantification of spines shown in the left panel (One-way ANOVA; **** *p* < 0.0001; ns = not significant). Data are depicted as mean ± standard error. NC: negative control. KD: knockdown.

Although it would be interesting to study further the upregulated genes linked to inflammatory processes, the majority of deregulated genes were decreased after the KD of *3222401L13Rik*, suggesting that this lncRNA could act as an activator of gene transcription under physiological conditions. The pathways linked to these downregulated genes indicate a potential function of *3222401L13Rik* in mediating neuronal support. Therefore, we decided to study in the context of this work the role of *3222401L13Rik* in gene activation and the regulation of neuronal support. In this regard, an important role of astrocytes is the removal of glutamate from the extracellular space, which is mainly mediated by glutamate transporter 1 (Glt-1) and glutamate aspartate transporter (Glast). We found that the levels of both transporters were reduced on the RNA (Figure 4A) and the protein level (Figure 4B) following the KD of *3222401L13Rik*. In line with this, the uptake of glutamate from the extracellular space was also impaired in primary astrocytes in the absence of *3222401L13Rik* (Figure 4C).

Another important feature of astrocytes is the ability to generate transient increases in intracellular calcium (Ca^2+^) levels in response to neuronal activity, which can induce the release of various gliotransmitters, e.g., glutamate, ATP, and GABA [32]. These gliotransmitters can, in turn, have a wide range of effects on neurons, such as the synchronization of action potential firing [33] or the regulation of synaptic vesicle release [34]. Pathological alterations of astrocytic Ca^2+^ dynamics have been described in different contexts, e.g., in aging and Alzheimer’s disease [35,36]. Therefore, we measured Ca^2+^ levels in response to adenosine triphosphate (ATP) stimulation and found that the increase in intracellular Ca^2+^ was reduced after the KD of *3222401L13Rik* compared to astrocytes treated with NC Gapmers (Figure 4D).

Since our previous results suggested that the lncRNA *3222401L13Rik* was important for maintaining neuronal support functions of astrocytes, we next asked whether the loss of *3222401L13Rik* in astrocytes affected neurons. Therefore, we cultured primary neurons alone or together with astrocytes treated with either control (+ NC astrocytes) or *3222401L13Rik* KD Gapmers (+ KD astrocytes). Then, we treated all groups with 100 µM glutamate and measured neuronal cell viability 3 h later. We found that the co-culture with NC astrocytes increased neuronal viability compared to the neuronal mono-culture, most likely due to the uptake of extracellular glutamate (Figure 4E). In contrast, the co-culture with KD astrocytes did not have these beneficial effects on cell survival (Figure 4E). Moreover, we assessed the effect of NC or KD astrocyte co-cultures on neuronal spine density. We found that only NC, but not KD, astrocytes increased the number of dendritic spines (Figure 4F), suggesting that the lncRNA *3222401L13Rik* indeed played an important role in maintaining neuronal support. This interpretation is further supported by electrophysiological measurements using Multielectrode array (MEA) assays (Figure S1).

### 2.5. The Regulation of Synaptic Support Genes Is Conserved in Human iPSC-Derived Astrocytes

**Figure 5:**
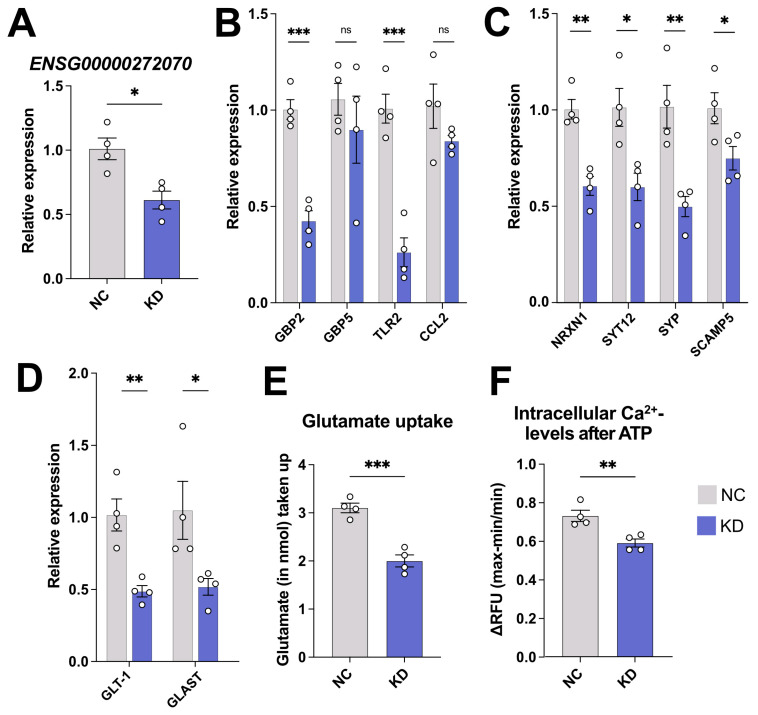
The synaptic support functions of *3222401L13Rik* are conserved in human astrocytes. (**A**) KD of *ENSG00000272070* in human iPSC-derived astrocytes (unpaired *t*-test; * *p* < 0.05). (**B**) Expression levels of interferon response genes after the KD of *ENSG00000272070* in human iPSC-derived astrocytes (unpaired *t*-test; *** *p* < 0.001; ns = not significant). (**C**) Expression levels of genes associated with synaptic support after the KD of *ENSG00000272070* in human iPSC-derived astrocytes (unpaired *t*-test; * *p* < 0.05; ** *p* < 0.01). (**D**) qPCR showing the expression of the glutamate transporters *GLT-1* and *GLAST* after the KD of *ENSG00000272070* in human iPSC-derived astrocytes (unpaired *t*-test; * *p* < 0.05; ** *p* < 0.01). (**E**) Glutamate uptake after the KD of *ENSG00000272070* (unpaired *t*-test; *** *p* < 0.001). (**F**) Increase in intracellular Ca^2+^ levels in response to ATP stimulation after the KD of *ENSG00000272070* (unpaired *t*-test; ** *p* < 0.01). Data are depicted as mean ± standard error. NC: negative control. KD: knockdown.

As *3222401L13Rik* has a human homolog, we next asked whether its function was conserved between species. We performed a KD of *ENSG00000272070* in human iPSC-derived astrocytes (Figure 5A) and measured via qPCR the levels of interferon response genes, which had been found to be upregulated in mouse astrocytes. However, *GBP2* and *TLR2* were significantly downregulated after the KD of *ENSG00000272070*, whereas the levels of *GBP5* and *CCL2* remained unchanged (Figure 5B). In contrast, we could recapitulate the findings from the mouse astrocytes regarding the downregulation of several genes linked to synaptic function (Figure 5C) and the two glutamate transporters *GLT-1* and *GLAST* in human iPSC-derived astrocytes (Figure 5D). Furthermore, also the uptake of extracellular glutamate and the increase in intracellular Ca^2+^ after ATP stimulation were impaired after the KD of *ENSG00000272070* (Figure 5E,F). These findings suggest a functional conservation of *3222401L13Rik* and *ENSG00000272070* regarding its role in maintaining the expression of genes important for neuronal support, whereas the upregulation of interferon response genes was specific for mouse astrocytes, also supporting our decision to focus our functional analysis on the role of *3222401L13Rik* in neuronal support.

### 2.6. 3222401L13Rik Interacts with Npas3 to Control Gene Expression

**Figure 6:**
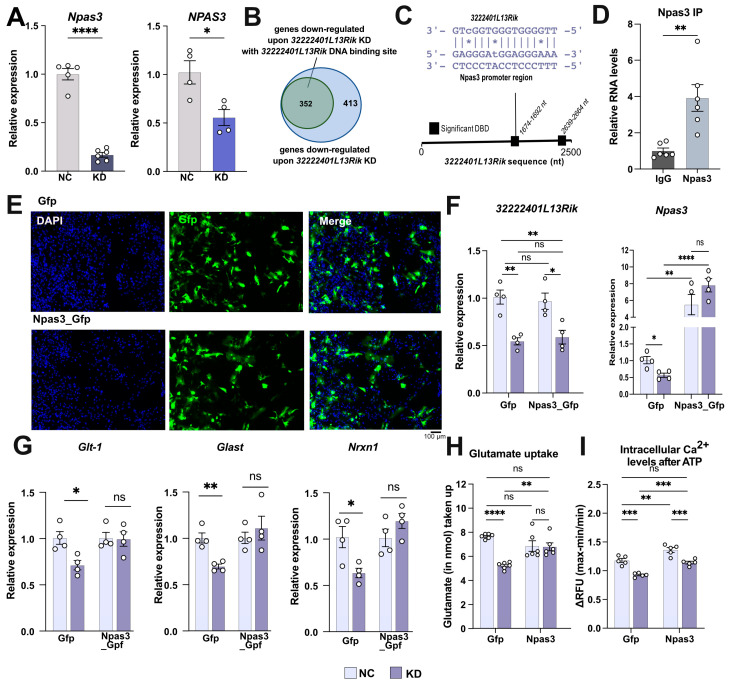
The overexpression of the interaction partner Npas3 can rescue molecular and functional changes induced by the loss of *3222401L13Rik*. (**A**) qPCR showing the expression levels of *Npas3/NPAS3* in mouse (left panel) and human iPSC-derived (right panel) astrocytes after the KD of *3222401L13Rik* (unpaired *t*-test; * *p* < 0.05, **** *p* < 0.0001). (**B**) Venn diagram showing the proportion of downregulated genes (352 out of 765) containing a promoter region that can bind *3222401L13Rik*. The Triplex Domain Finder tool [39] was used to identify promoter regions of the downregulated genes that have a binding motif for *3222401L13Rik*. (**C**) Scheme depicting the significant DNA binding domains (DBD) in the sequence of *3222401L13Rik* determined using the Triplex Domain Finder tool and the sequence motifs where *3222401L13Rik* binds to the promoter of Npas3. (**D**) RNA immunoprecipitation for Npas3, followed by qPCR for *3222401L13Rik* in mouse primary astrocytes (unpaired *t*-test; ** *p* < 0.01). (**E**) Representative immunofluorescence images showing the transfection of primary astrocytes with Gfp- or Gfp-Npas3-overexpression plasmids. Scale bar: 100 µm. (**F**) Expression levels of *3222401L13Rik* and Npas3 after the simultaneous KD of *3222401L13Rik* and overexpression of Npas3 in primary astrocytes (One-way ANOVA; * *p* < 0.05; ** *p* < 0.01; **** *p* < 0.0001; ns = not significant). (**G**) Expression levels of *Glt-1*, *Glast,* and *Nrxn1* after the simultaneous KD of *3222401L13Rik* and overexpression of Npas3 in primary astrocytes (One-way ANOVA; * *p* < 0.05; ** *p* < 0.01; ns = not significant). (**H**) Glutamate uptake of primary astrocytes after the simultaneous KD of *3222401L13Rik* and overexpression of Npas3 (One-way ANOVA; ** *p* < 0.01; **** *p* < 0.0001; ns = not significant). (**I**) Increase in intracellular Ca^2+^ levels in response to ATP stimulation after the simultaneous KD of *3222401L13Rik* and overexpression of Npas3 (One-way ANOVA; ** *p* < 0.01; *** *p* < 0.001; ns = not significant). Data are depicted as mean ± standard error. NC: negative control. KD: knockdown.

Among the most significantly downregulated genes identified from the RNA sequencing data was the transcription factor Neuronal PAS Domain Protein 3 (Npas3) (Table S3). We confirmed this finding by qPCR in primary mouse and human iPSC-derived astrocytes (Figure 6A). Npas3 is known as an important transcription factor in astrocytes that mediates the expression of genes involved in brain development and synapse function [37]. This study also demonstrated that an astrocyte-specific knockout of Npas3, similar to our data, causes deficits in synaptic density and complexity, along with behavioral abnormalities in mice. Additionally, Npas3 target genes were found to be significantly enriched in genes associated with schizophrenia, autism, and intellectual disability [38]. Based on these findings, we hypothesized that the effects observed after the knockdown (KD) of 3222401L13Rik might, at least in part, be mediated by the deregulation of Npas3.

lncRNAs are known to bind to DNA and form triple helices, acting as scaffolds to facilitate interactions between proteins, RNA, and DNA [39]. We employed the Triplex Domain Finder tool [39] to assess how many downregulated genes could potentially bind *3222401L13Rik* via their promoter region. This analysis revealed that 46% (352 out of 765 transcripts) exhibited *3222401L13Rik*-binding regions (Figure 6B) (Table S5) that were localized toward the 3′ end of *3222401L13Rik* (Figure 6C), including the potential binding site for the *Npas3* promoter (Figure 6C), further supporting our biochemical data, suggesting that *3222401L13Rik* could directly bind to the promoter of *Npas3*. It is known that *Npas3* expression is regulated by autoregulation, where the Npas3 protein binds to its own gene promoter to drive its expression [38]. Therefore, we hypothesized that *3222401L13Rik* might interact with both the *Npas3* promoter and Npas3 itself to regulate its expression. Indeed, we observed a direct interaction between *3222401L13Rik* and Npas3, as demonstrated by RNA immunoprecipitation followed by qPCR (Figure 6D).

These data support a model in which *3222401L13Rik* positively regulates *Npas3* expression, likely by promoting positive autoregulation. Loss of *3222401L13Rik* reduces Npas3 levels. To test whether overexpression of *Npas3* could rescue the functional impairments observed after *3222401L13Rik* knockdown, we electroporated primary mouse astrocytes with plasmids containing *Npas3* and *Gfp* (Npas3_GFP) under the control of the astrocyte-specific Gfap promoter—to monitor successful transfection—or only Gfp as a control (Gfp_control) (Figure 6E). We then combined this with the transfection of Gapmers to knock down *3222401L13Rik* or control Gapmers. While *3222401L13Rik* knockdown reduced the expression of Npas3 in astrocytes transfected with Gfp (Figure 6F), we observed significant overexpression of Npas3 in astrocytes electroporated with the Npas3_Gfp construct (Figure 6F). Importantly, knockdown of *3222401L13Rik* did not affect *Npas3* expression in this overexpression setting (Figure 6F), allowing us to test if Npas3 overexpression could rescue the phenotypes associated with loss of *3222401L13Rik* function.

Consistent with previous data (see Figure 3F and Figure 4A), knockdown of *3222401L13Rik* reduced the levels of *Glt-1, Glast*, and *Nrxn1* (Figure 6G) in astrocytes electroporated with the GFP plasmid. However, overexpression of Npas3 reinstated physiological expression levels of all three genes despite the knockdown of *3222401L13Rik* (Figure 6G). Similar observations were made for glutamate uptake and intracellular calcium levels. Consistent with previous data (see Figure 4C), glutamate uptake was impaired upon *3222401L13Rik* knockdown in astrocytes transfected with Gfp (Figure 6G), while *Npas3* overexpression could rescue this phenotype (Figure 6H). Finally, we measured intracellular Ca^2+^ levels following ATP stimulation. Similar findings were observed for intracellular Ca^2+^ levels upon ATP stimulation (Figure 6I).

In summary, these data suggest that *3222401L13Rik* positively regulates and fine-tunes the expression of Npas3 and that the phenotypes observed upon 3222401L13Rik knockdown are, at least in part, mediated by loss of Npas3 function.

## 3. Discussion

In this study, we identify *3222401L13Rik* as a glial-enriched lncRNA with elevated expression levels in astrocytes of aged mice. To our knowledge, the function of this lncRNA has not been studied in any cellular system before. However, *3222401L13Rik* was found to be upregulated in two distinct RNA-seq datasets related to brain aging. Specifically, upregulation of *3222401L13Rik* was observed in ApoD knockout mice, which are characterized by premature brain aging, when bulk cortical tissue was analyzed via RNA-seq [40]. Similarly, upregulation of *3222401L13Rik* was detected in the hippocampi of young and old mice, as characterized by both bulk and single-cell RNA-seq [41]. Although neither study specifically highlights *3222401L13Rik*, these findings support our observation and suggest a role for *3222401L13Rik* in brain aging.

We found that *3222401L13Rik* was highly expressed in glial cells compared to neurons, which aligned with previous research showing that lncRNAs often exhibited tissue-specific expression patterns [19]. The age-associated upregulation of *3222401L13Rik* was specific to astrocytes. While we cannot yet explain the underlying molecular mechanisms, it is known that stimuli such as environmental enrichment, aging, and neurodegenerative diseases lead to cell-type-specific changes in gene expression in the brain [42,43]. Moreover, astrocytes play a crucial role in brain aging, and recent studies suggest that transcriptional changes in astrocytes are a central mechanism for promoting resilience against cognitive decline in Alzheimer’s disease [44]. Therefore, we decided to study the function of *3222401L13Rik* in astrocytes. It is likely that *3222401L13Rik* also regulates important cellular processes in other glial cells, such as microglia and oligodendrocytes, which we plan to address in future research.

We found that *3222401L13Rik* is primarily expressed in the nucleus, which aligns with the known role of lncRNAs in gene expression control [13]. Consistent with this, we observed that the knockdown of *3222401L13Rik* in primary mouse astrocytes led to significant changes in gene expression, including upregulation of inflammatory processes and a downregulation of genes associated with neuronal support functions. This finding is consistent with previous studies showing that lncRNAs can have profound effects on astrocytic gene expression control [22,25].

Strikingly, using RNAscope, we observed that 3222401L13Rik was localized to two to three nuclear foci per astrocyte. This kind of signal has also been observed in other studies investigating lncRNAs [45,46]. Moreover, Cabili et al. visualized the localization of more than 60 lncRNAs and identified different categories of cellular localization patterns, ranging from bright sub-nuclear foci—similar to the signal we observed for 3222401L13Rik—to a dispersed nuclear or cytoplasmic distribution [47].

This study further suggests that lncRNAs showing bright nuclear foci are involved in chromatin regulation and are localized to sites of active transcription [47], which aligns with our finding that 3222401L13Rik regulates the expression of synapse-associated genes. Additionally, lncRNAs are critical for the formation of nuclear condensates with specific functions, such as nuclear speckles [13] which could also explain the localization of lncRNA molecules to nuclear foci. However, further studies will be necessary to elucidate the exact localization of 3222401L13Rik within the nucleus of astrocytes.

In human iPSC-derived astrocytes, knockdown of the homologous lncRNA *ENSG00000272070* also decreased the expression of genes linked to neuronal support functions. However, no increased expression of genes linked to inflammatory processes was observed. It is important to note that these data are based on qPCR analysis of selected transcripts. Nonetheless, recent studies comparing the transcriptomic signatures of astrocyte reactivity in rodent and human models have identified both commonalities and species-specific gene signatures [48,49]. While there are commonalities, distinct gene expression programs have been identified. For instance, human astrocytes are more susceptible to oxidative stress and demonstrate stronger activation of the antigen presentation pathway under inflammatory conditions. In contrast, mouse astrocytes show unique features, such as the induction of a molecular program for neural repair under hypoxia [44]. These observations underscore the fact that species-specific differences are to be expected when comparing mouse and human astrocytes. Thus, the results of our study may reflect partially divergent regulation of astrocyte reactivity between these species, which is an important consideration for translating findings from rodent models to humans.

Interestingly, previous studies using postmortem human brain tissue from AD patients have suggested a modest increase in proinflammatory genes in astrocytes and, more strikingly, a significant decrease in the expression of homeostatic genes involved in synapse regulation. Based on these findings, we further characterized the role of *3222401L13Rik* and *ENSG00000272070* in neuronal support functions. However, we acknowledge the importance of elucidating the role of these lncRNAs in inflammatory processes in future research. The use of alternative, more complex human models, such as brain organoids, could also provide more insight into similarities and differences between the mouse and human lncRNA with regard to neuronal support and the regulation of inflammatory pathways.

Our findings demonstrate that *3222401L13Rik*, as well as its human homolog, play crucial roles in maintaining the expression of several genes critical for neuronal and synaptic support within astrocytes. These include the glutamate transporters Glt1 and Glast, as well as genes controlling synaptic plasticity, such as Nrxn1. Consistent with this, the knockdown of *3222401L13Rik* impaired glutamate uptake and regulation of intracellular Ca^2^⁺ levels in astrocytes. Additionally, astrocytes lacking *3222401L13Rik* were unable to efficiently support neurons, as evidenced by altered neuronal network plasticity and decreased neuronal spine density. Although specific data for *3222401L13Rik* or *ENSG00000272070* are currently lacking, previous studies have shown that lncRNAs could regulate key cellular functions in astrocytes, including glutamate metabolism, calcium signaling, and neuronal network homeostasis [22,25,50,51].

One mechanism by which lncRNAs control gene expression is through interactions with DNA, particularly in gene-promoter regions, and with proteins, thereby orchestrating the function of transcriptional regulators [13]. Bioinformatic analysis revealed that half of the transcripts downregulated upon *3222401L13Rik* knockdown contained binding regions for *3222401L13Rik* in their promoter regions, suggesting that these might be direct targets. Future work will be required to confirm these DNA–lncRNA interactions experimentally. Among these potential targets is Npas3, a transcription factor associated with neuropsychiatric disorders characterized by impaired neuronal plasticity, such as schizophrenia [52,53,54,55,56]. Our study shows that *3222401L13Rik* regulates *Npas3*, which is noteworthy since *Npas3* is highly expressed in astrocytes, and its deficiency induces synaptic deficits in neurons [37]. Moreover, the affected pathways and gene networks identified in this study closely resemble the downregulated genes and pathways observed after the knockdown of *3222401L13Rik*, including terms like “synapse organization”, “neurotransmitter transport”, and “synapse assembly”. Accordingly, we demonstrate that overexpression of *Npas3* can rescue both molecular and functional deficits resulting from the knockdown of *3222401L13Rik*. These data strongly suggest that *3222401L13Rik* orchestrates the regulation of astroglia function—at least in part—via Npas3.

Taking into account that *3222401L13Rik* expression increases in the aging brain and that its knockdown impairs astrocytes’ ability to perform critical neuronal support functions, it can be speculated that altered *3222401L13Rik* expression may represent an adaptive mechanism aimed at mitigating ongoing network imbalances and functional alterations associated with aging. This would further imply that increasing *3222401L13Rik* expression or strengthening the interaction of *3222401L13Rik* and Npas3 in early aging processes or at the onset of neurodegenerative diseases such as AD could be a potential clinical option to delay the onset of neuronal dysfunction. However, further studies are needed to determine whether modulating *3222401L13Rik* and *ENSG00000272070* levels can indeed enhance astrocytic function and neuronal support during aging and neurodegeneration. To this end, preclinical studies using animal models or human iPSC-derived cellular systems that mimic neuronal dysfunction or neurodegeneration should explore approaches to increase *3222401L13Rik* expression in astrocytes, such as CRISPRa-based methods.

Our study has several limitations that should be acknowledged. First, increasing evidence reveals distinct astrocyte subtypes within and between brain regions [57]. Since our study focused on the hippocampus, it would be important to investigate the role of *3222401L13Rik* and *ENSG00000272070* in other brain regions. Additionally, analyzing the expression of these lncRNAs in postmortem human brains from both young and old individuals, as well as those with cognitive diseases, would be crucial. Ideally, this should be complemented by similar analyses in animal models, allowing us to determine if *3222401L13Rik* expression is a compensatory mechanism aimed at ameliorating the detrimental effects of brain aging. This is particularly interesting given recent data suggesting a key role of astrocytes in mediating resilience to cognitive decline [44]. It would also be valuable to study the functional role of *3222401L13Rik* in an in vivo system. The knockdown of *3222401L13Rik* in wild-type mice and its overexpression in disease models could provide important insights critical for the preclinical development of RNA-based cognitive enhancers. However, preclinical research involving mice is becoming increasingly difficult in the European Union, particularly in Germany, and we are unable to perform such experiments within a reasonable time frame. Finally, our dataset suggests several other lncRNAs that are deregulated in the aging mouse brain and have human homologs. In addition to *Neat1*, which is well-studied for its role in brain function and disease [25,58,59,60,61], we identified *A330023F24Rik*, *Lncpint*, *Gm12689*, *Mir124-2hg*, *B130024G19Rik*, *C430049B03Rik*, and *Gm45847* as being deregulated in the aging mouse hippocampus and having human homologs. *A330023F24Rik* is deregulated in Duchenne muscular dystrophy [62] and in response to optic nerve injury [63]. *Lncpint*, a neuron-enriched lncRNA, is increased in various neurodegenerative diseases, including Alzheimer’s disease (AD), Parkinson’s disease, and Huntington’s disease, and functional studies suggest a protective role since the loss of Lncpint exacerbates pathology [64], which is similar to our data on *3222401L13Rik. Mir124-2hg* is also a neuron-enriched lncRNA with a reported role in synaptic vesicle recycling [65] that warrants further analysis in neurons. The human homolog of *B130024G19Rik, NR2F2-AS1,* has been studied in various cancers, as has *C430049B03Rik* and its human homolog *MIR503HG*.

In summary, our study identifies the novel lncRNA *3222401L13Rik* as crucial in regulating astrocytic gene expression through its interaction with the transcription factor Npas3. The observed upregulation of *3222401L13Rik* suggests that it acts as an adaptive mechanism in astrocytes to support neuronal function during aging. Given that age-associated memory decline and the pathogenesis of neurodegenerative diseases are characterized by distinct molecular and cellular phases [66], strategies aimed at enhancing *3222401L13Rik* function could offer promising avenues for developing targeted therapies for cognitive diseases.

## 4. Materials and Methods

### 4.1. Animals

All animal experiments were approved by the local Animal Welfare Office of Goettingen University and the Lower Saxony State Office for Consumer Protection and Food Safety. Three- and 16-month-old male C57/BL6 and pregnant CD-1 mice were obtained from Janvier Labs. Animals were housed in standard cages with a 12-h dark and light cycle. Water and food were provided ad libitum.

### 4.2. Sorting of Neuronal and Non-Neuronal Nuclei from Mouse Brain

Young and old C57/BL6 mice were sacrificed using pentobarbital or CO_2_. Brains were quickly removed, and the CA1 region of the hippocampus was dissected using a dissection microscope. Brain tissue was flash-frozen and kept at −80 °C until further use. Nuclei isolation, NeuN-staining, and FANS-sorting were performed as previously described [67,68]. After sorting, Trizol LS (ThermoFisher, Waltham, MA, USA) was added, and RNA was isolated, as described below.

### 4.3. Primary Astrocyte Culture

For the generation of primary astrocyte cultures, a protocol was used that allowed for the cultivation of astrocytes that were less reactive compared to cultures permanently containing fetal bovine serum (FBS) [69]. Briefly, postnatal day 0 to 2 pups were sacrificed by decapitation; the brains were quickly taken out, and the meninges were removed. Cortices and hippocampi were dissociated using 0.05% Trypsin–Ethylenediaminetetraacetic acid (EDTA) (Gibco, Waltham, MA, USA), and the resulting cells were seeded into T75 flasks coated with 0.5 mg/mL Poly-D-lysine (PDL) (MerckMillipore, Burlington, MA, USA) and kept in DMEM containing 1% penicillin–streptomycin and 10% FBS (all Gibco) in a humidified incubator with 5% CO_2_ at 37 °C. After 7–8 days, the flasks were shaken at 160 rpm for 6 h to remove non-astrocytic cells. Astrocytes were removed from the flasks using 0.25% Trypsin–EDTA (Gibco), centrifuged, counted, and seeded at a density of 15,000 cells/cm^2^ on pre-coated cell culture dishes. Cells were kept in Neurobasal Plus Medium containing 2% B27 Plus supplement, 1× GlutaMax, and 1% penicillin–streptomycin (NB+) (all Gibco) with 5 ng/mL heparin-binding EGF-like growth factor (HB-EGF) (Sigma-Aldrich, Schnelldorf, Germany) until experiments were performed.

### 4.4. Primary Neuron Culture

Pregnant CD-1 mice were sacrificed using pentobarbital on embryonic day 17. The embryos were taken out, and their brains were removed. After dissection of the meninges, cortices and hippocampi were dissociated using the Papain Dissociation System (Worthington Biochemical Corporation, OH, USA), as described by the manufacturer’s instructions. Finally, cells were resuspended in NB+, counted, and seeded at a density of 120,000 cells/cm^2^ for glutamate treatment experiments or 60,000 cells/cm^2^ for spine analysis on cell culture dishes pre-coated with 0.5 mg/mL PDL or 0.1% polyethylenimine (PEI).

On day 14 in vitro (DIV), astrocytes cultured in co-culture inserts (Greiner, Kremsmünster, Austria) were added to neurons, and experiments were performed on DIV17.

### 4.5. Human iPSC-Derived Astrocytes

Human induced pluripotent stem cell (iPSC)-derived astrocytes were obtained from Ncardia. Cells were shipped and stored in liquid nitrogen until use. Cells were thawed and cultured according to the manufacturer’s instructions. Transfections and experiments were performed at least one week after thawing.

### 4.6. Antisense LNA Gapmers

Custom Antisense LNA Gapmers directed against *3222401L13Rik* and *ENSG00000272070,* as well as negative controls, were designed by and obtained from Qiagen. Sequences were aligned to the mouse genome to minimize the risk of off-target effects. The following sequences were used:

NC: GCTCCCTTCAATCCAA;

*3222401L13Rik*: AGCTTGGTCATTTGAT;

*ENSG00000272070*: GGACTTCTTCCTCTGT.

Primary and iPSC-derived astrocytes were transfected with 50 nM Gapmers using Lipofectamine RNAiMax (ThermoFisher) according to the manufacturer’s instructions. Primary astrocytes were transfected on DIV12, and functional experiments were performed 48 h later. iPSC-derived astrocytes were transfected at least one week after thawing and collected after 48 h.

### 4.7. Npas3 Overexpression

Expression plasmids encoding the open reading frame for Npas3-Gfp or Gfp only under the control of the glial fibrillary acidic protein (Gfap)-promoter were designed by and purchased from VectorBuilder (Neu-Isenburg, Germany). Primary astrocytes were transfected on DIV7 before seeding using the Neon Nxt electroporation device (ThermoFisher). A total of 100,000 cells were electroporated using 0.25 µg plasmid and 25 nM Antisense LNA Gapmers (1300 V, 20 ms, 2 pulses) and seeded in pre-coated 24-well plates. Experiments were performed 72 h after transfection.

### 4.8. Glutamate Uptake

To measure the amount of glutamate taken up from the extracellular space, primary and iPSC-derived astrocytes were pre-incubated with Hank’s Buffered Salt Solution (HBSS) for 10 min at 37 °C. Then, the supernatant was aspirated, and 100 µM glutamate in HBSS was added. After 1 h for primary astrocytes and 3 h for iPSC-derived astrocytes, the supernatant was collected, and the remaining glutamate was measured using the Glutamate-Glo™ Assay (Promega, Madison, WI, USA) according to the manufacturer’s instructions. Luminescence was recorded with a FLUOstar^®^ Omega plate reader (BMG, Ortenberg, Germany).

### 4.9. Measurement of Intracellular Ca^2+^ Levels

Primary mouse and human iPSC-derived astrocytes were incubated with Fluo-4 calcium assay reagent (Fluo-4 Direct™ Calcium-Assay-Kit, ThermoFisher) for 30 min at 37 °C before recording baseline fluorescence levels (RFU_Min_) using a plate reader. Then, 100 µM ATP was added, and the plate was measured immediately again (RFU_Max_). The difference in relative fluorescence units (ΔRFU) was calculated using the following formula: ΔRFU = (RFU_Max_ − RFU_Min_)/RFU_Min_. As a blank, wells containing no cells were used.

### 4.10. Collection of Astrocyte-Conditioned Medium (ACM)

For the treatment of neurons with ACM, medium from NC or KD astrocytes was collected 48 h after treatment, filtered using a 0.22 µm filter, and stored at −80 °C until use. On DIV14, primary neurons were treated with ACM that was diluted 1:1 with fresh NB+.

### 4.11. Glutamate Treatment of Primary Neurons

Primary neurons cultured either alone or together with NC or KD astrocytes or with ACM from NC or KD astrocytes were treated with 100 µM glutamate for 15 min at 37 °C. Then, the medium was removed, and fresh NB+ was added. Three hours later, cell viability was assessed using PrestoBlue (ThermoFisher).

### 4.12. Multielectrode Array

Multielectrode array (MEA) recordings were performed in the Maestro system (Axion), as previously described [70]. ACM from NC or KD astrocytes was added to DIV14, and recordings were performed twice a day until DIV17.

### 4.13. Dendrite and Spine Analysis

Spines from primary neurons cultured either alone or together with NC or KD astrocytes or with NC or KD ACM were analyzed as previously described [71]. Briefly, cells were fixed using 2% paraformaldehyde (PFA), and dendrites and spines were labeled using the dye 1,1′-dioctadecyl-3,3,3′,3′-tetramethylindocarbocyanine perchlorate (Dil) (ThermoFisher). Dendrite length and the number of spines were measured using ImageJ software (Version 2.9.0/1.53t; National Institutes of Health (NIH), Bethesda, MD, USA).

### 4.14. Sorting of Astrocytes, Oligodendrocytes, and Microglia Using MACS

Three- and 16-month-old C57/BL6 mice were sacrificed using pentobarbital or CO_2_. The brains were quickly isolated, and the meninges were removed by carefully rolling the brains over Whatman paper. Then, the tissue was dissociated using the adult brain dissociation kit (cat. no. 130-107-677, Miltenyi, Bergisch Gladbach, Germany) according to the manufacturer’s protocol with minor modifications. Briefly, the minced tissue was incubated with the enzyme mix for 30 min at 37 °C in a water bath and gently triturated after 5, 15, and 25 min. Then, the suspension was applied to 40 µm cell strainers, and the steps for red blood cell and debris removal were performed. Antibody labeling was performed using the following antibodies: Anti-O4 microbeads (1:40, cat. no. 130-094-543); Anti-ACSA2 microbeads (1:10, cat. no. 130-097-678); and Anti-Cd11b microbeads (1:10, cat. no. 130-093-634). Oligodendrocytes, astrocytes, and microglia were isolated using the MACS technique. The purity of the resulting populations was assessed using qPCR.

### 4.15. RNAscope Combined with Immunofluorescence

To stain for both *3222401L13Rik* and the astrocyte marker Gfap, we combined RNAscope Fluorescent Multiplex assays (Acdbio, Minneapolis, MN, USA) and immunofluorescence according to the manufacturer’s protocol for fresh frozen tissue. Briefly, C57/BL6 mice were sacrificed using pentobarbital or CO_2_, and the brains were quickly removed, embedded in optimal cutting temperature (OCT, Toronto, ON, Canada) compound, flash-frozen in liquid nitrogen, and stored at −80 °C until use. The 18 µm tissue sections were prepared using a cryostat (Leica, Wetzlar, Germany). Then, the tissue sections were fixed using 10% neutral buffered formalin, dehydrated, and treated with hydrogen peroxide before the overnight incubation with the primary antibody anti-Gfap (rabbit, Abcam, Cambridge, UK; 1:250). On the next day, after a post-primary fixation, *3222401L13Rik* was labeled using the RNAscope^®^ Multiplex Fluorescent Reagent Kit v2 (Acd Bio, Newark, CA, USA) with probes designed to target the lncRNA and TSA Plus Cyanine 5 (Akoya Biosciences, Marlborough, MA, USA; 1:750) for detection. Finally, the secondary antibody (Alexa Fluor™ 555 goat anti-rabbit secondary antibody; 1:1000, ThermoFisher) and DAPI (Sigma-Aldrich) were applied, and the slides were mounted using Prolong Gold Antifade Reagent (ThermoFisher). Confocal images were acquired within a week after staining.

### 4.16. Imaging

Fluorescent images were taken with a Leica dmi8 microscope, confocal images with the same microscope fitted with a STEDycon STED/Confocal (Abberior, Göttingen, Germany) in confocal mode, with a 63× or 100× oil immersion objective.

### 4.17. Cytoplasmic and Nuclear Fractionation

Primary astrocytes were harvested on DIV14 using 0.25% Trypsin-EDTA, and the resulting pellets were washed with PBS. To isolate the cytoplasmic fraction, 500 µL EZ prep lysis buffer (Sigma-Aldrich) supplemented with RNase inhibitor (Promega, Madison, WI, USA) was added and incubated on ice for 7 min. The samples were centrifuged, and the supernatant containing the cytoplasmic fraction was collected. The nuclear pellet was washed with EZ prep lysis buffer and resuspended in 1.5 mL phosphate-buffered saline (PBS) supplemented with 0.5% bovine serum albumin (BSA, Irving, TX, USA) (CellSignal, Danvers, MA, USA), RNase inhibitor and protease inhibitor (Roche, Basel, Switzerland). After centrifugation, the supernatant was aspirated, leaving 250 µL of liquid in the tube. TRIzol™ LS Reagent (ThermoFisher) was added to both the cytoplasmic and nuclear fractions, and RNA was extracted as described in the next section.

### 4.18. RNA Isolation

Cells were lysed using TRI reagent (Sigma-Aldrich), and subsequent RNA extraction was performed using the RNA clean and concentrator-5 kit (Zymo Research, Irvine, CA, USA) according to the manufacturer’s instructions.

RNA concentrations were measured with Nanodrop or Qubit (both ThermoFisher). RNA quality was assessed using a Bioanalyzer (Agilent Technologies, Santa Clara, CA, USA) before subjecting the samples to total RNA sequencing.

### 4.19. cDNA Synthesis and qPCR

Transcriptor cDNA first strand Synthesis Kit (Roche) and random hexamer primers were used to prepare cDNA from 20–800 ng RNA starting material according to the manufacturer’s instructions. Synthesized cDNA was diluted to a concentration of 1 ng/µL with nuclease-free water. The qPCR reactions were prepared using LightCycler 480 SYBR Master Mix (Roche) and run in duplicates in a LightCycler 480 (Roche). Primer sequences are listed in Table S6. Analysis was performed using the 2^−DDCt^ method [72]. ncRNA genes were normalized to the expression of *18S*, while *Gapdh* was used for the normalization of protein-coding genes.

### 4.20. Library Preparation and Total RNA Sequencing

The SMARTer Stranded Total RNA Sample Prep Kit—HI Mammalian kit (Takara Bio, San Jose, CA, USA)—with 300 ng RNA as input was used for library preparation according to the manufacturer’s instructions. Thirteen cycles were applied for library amplification, and the quality of the libraries was assessed using a Bioanalyzer. Then, the multiplexed libraries were sequenced in a NextSeq 2000 (Illumina, San Diego, CA, USA) with a 50 bp single-read configuration.

### 4.21. Bioinformatic Analysis

Raw reads were processed and demultiplexed using bcl2fastq (v2.20.2). Quality control of raw sequencing data was performed using FastQC (v0.11.5). Reads were aligned to the mouse (mm10) genome with the STAR aligner (v2.5.2b), and feature Counts (v1.5.1) were used to generate read counts. Differential gene expression was performed with DESeq2 (v1.38.3) [73]. For this, normalized read counts were used, and correcting for unwanted variation detected by RUVSeq (v1.32.0) [74] was applied. GO term analysis was performed using clusterProfiler (v4.6.0) [31].

### 4.22. Western Blot

Primary astrocytes were harvested and lysed with RIPA buffer (ThermoFisher) containing 1× protease inhibitor (Roche). Protein concentration was measured with Pierce BCA Protein Assay Kit (ThermoFisher), and 20 µg protein was used per well of a 4–20% Mini-PROTEAN^®^ TGX™ Precast Protein Gel (Bio-Rad, Hercules, CA, USA). Protein denaturation was carried out using 8 M Urea in 1× Laemmli Sample Buffer (Bio-Rad) for 60 min at 40 °C. The gels were run at 90 V for 15 min and 120 V for 50 min. Transfer to low-fluorescence PVDF membranes was performed with the Trans-Blot Turbo Transfer System (both from Bio-Rad).

Then, membranes were blocked with 5% BSA in PBS + 0.1% Tween-20 (PBS-T), followed by overnight incubation with the following primary antibodies: anti-Eaat1 (rabbit, Abcam; 1:2500); anti-Eaat2 (rabbit, Abcam; 1:800); and anti-Gapdh (mouse, ThermoFisher; 1:4000) (Table S7). On the next day, the membranes were washed three times with PBS-T and incubated with the corresponding secondary antibodies (IRDye, LI-COR; 1:10,000) for 1 h at room temperature. After three subsequent washes with PBS-T, the membranes were imaged with an Odyssey DLx (LI-COR), and the blots were analyzed with ImageJ software.

### 4.23. RNA Immunoprecipitation (RNA-IP)

Primary astrocytes were cultured in 15 cm dishes and harvested on DIV14. Cell pellets were resuspended in fractionation buffer (10 mM Tris-HCl, 10 mM NaCl, 3 mM MgCl2, 0.5% Nonidet P-40 (NP-40), 1mM DTT, 100 units/mL RNase inhibitor, 1x protease inhibitor) and incubated on ice for 10 min with occasional pipetting to lyse cells. Then, the samples were centrifuged at 1000× *g*, 5 min, at 4 °C, and the supernatant containing the cytoplasmic fraction was transferred into fresh tubes. The pellet was washed with TSE buffer (10 mM Tris, 300 mM sucrose, 1 mM EDTA, 0.1% NP-40, 1 mM DTT, 100 units/mL RNase inhibitor, 1× protease inhibitor), resuspended in fresh TSE buffer, transferred to bioruptor tubes, and sonicated in a Bioruptor Plus (both Diagenode, Denville, NJ, USA) for ten cycles (30 s on, 30 s off). Afterward, the samples were incubated on ice for 20 min with occasional vortexing and centrifuged at 14,500× *g* for ten minutes. The supernatant was transferred to a fresh tube, and the protein concentration of both fractions was determined, as described above. Then, all samples were flash-frozen and stored at −80 °C until further use.

For the RNA-IP, 500 µg protein from the nuclear lysate per sample was pre-cleared using 25 µL Pierce™ Protein A/G magnetic beads (ThermoFisher) for 1 h at 4 °C. Simultaneously, 1.5 µg Npas3 antibody (rabbit, ThermoFisher) or 1.5 µg IgG isotype control (rabbit, ThermoFisher) per sample were incubated with 50 µL Protein A/G magnetic beads for two hours at RT, followed by a wash with RNA-IP buffer (50 mM Tris-HCl, 100 mM NaCl, 32 mM NaF, 0.5% NP-40). When pre-clearing of the samples was performed, samples were put on a magnetic rack to separate the beads from the lysate; 10% of each sample was collected to serve as an input control, and the remaining liquid was added to the beads. The samples were incubated overnight at 4 °C with mixing. On the next day, the beads were washed five times with RNA-IP buffer and then resuspended in a proteinase K buffer containing proteinase K (Qiagen, Hilden, Germany), followed by an incubation at 37 °C for one hour. The beads were again separated using a magnetic rack, and the supernatant was transferred to fresh tubes. RNA was extracted using the RNA clean and concentrator-5 kit (Zymo Research) according to the manufacturer’s instructions.

### 4.24. Statistical Analysis

For statistical analysis, GraphPad Prism version 9 was used. All graphs are shown as mean ± standard error unless stated otherwise. A two-tailed unpaired *t*-test or a one-way ANOVA with Tukey’s post hoc test were applied for data analysis. Enriched gene ontology and pathway analysis was performed using Fisher’s exact test followed by a Benjamini–Hochberg correction.

## Figures and Tables

**Table 1:** Characteristics of lncRNAs deregulated during aging in Neu– nuclei.

lncRNA Name	log2FC 3 vs. 16 Months	*p*-adj	Log2FC Neu–vs. NeuN+	*p*-adj	Conserved	Human Homolog
**Upregulated in 16-month-old mice**						
**Neat1**	**0.36**	**1.08 × 10^−5^**	**7.82**	**0.00 × 10^+00^**	**Yes**	**Neat1**
Gm42756	0.40	2.36 × 10^−5^	9.48	3.67 × 10^−83^	No	
A330023F24Rik	0.31	1.58 × 10^−4^	−1.90	2.71 × 10^−112^	Yes	MIR29B2CHG
9330111N05Rik	0.58	4.96 × 10^−7^	4.97	7.96 × 10^−198^	No	
Lncpint	0.47	1.32 × 10^−4^	−1.19	1.15 × 10^−25^	Yes	LINC-PINT
**3222401L13Rik**	**0.31**	**1.44 × 10^−3^**	**4.31**	**2.50 × 10^−177^**	**Yes**	**ENSG00000272070**
E230029C05Rik	0.31	4.72 × 10^−2^	1.60	4.67 × 10^−43^	No	
7630403G23Rik	0.86	3.96 × 10^−13^	9.74	4.32 × 10^−33^	No	
Gm43242	0.31	1.41 × 10^−2^	8.54	4.00 × 10^−53^	No	
B230216N24Rik	0.51	6.73 × 10^−4^	−0.97	1.99 × 10^−19^	No	
AU020206	0.47	1.31 × 10^−3^	7.18	1.94 × 10^−51^	No	
C130073E24Rik	0.54	1.45 × 10^−4^	−2.18	2.03 × 10^−77^	No	
Gm5086	0.41	1.50 × 10^−2^	3.60	4.94 × 10^−41^	No	
A530053G22Rik	2.11	1.47 × 10^−29^	7.10	4.60 × 10^−21^	No	
Gm12689	0.44	2.16 × 10^−2^	1.72	1.83 × 10^−15^	Yes	ENSG00000237163
Gm15802	0.45	4.73 × 10^−2^	1.78	1.13 × 10^−9^	No	
Gm10069	0.77	1.78 × 10^−3^	0.99	3.31 × 10^−3^	No	
**Downregulated in 16-month-old mice**						
Mir124-2hg	−0.41	2.67 × 10^−3^	−2.74	9.96 × 10^−267^	Yes	MIR124-2HG
Gm4221	−1.19	4.48 × 10^−12^	3.78	1.11 × 10^−48^	No	
Gm10530	−0.46	3.75 × 10^−2^	1.48	2.29 × 10^−16^	No	
B130024G19Rik	−0.53	3.22 × 10^−2^	−1.04	4.10 × 10^−10^	Yes	NR2F2-AS1
**C430049B03Rik**	**−0.82**	**6.31 ×** 10**^−5^**	**9.66**	**2.49 × 10^−28^**	**Yes**	**MIR503HG**
Gm13270	−0.55	4.66 × 10^−2^	1.77	1.65 × 10^−13^	No	
Gm45847	−0.79	4.20 × 10^−2^	−0.88	6.30 × 10**^−4^**	Yes	TNRC6A

This table displays the 17 lncRNAs that are significantly altered when comparing NeuN− nuclei from 3-month-old versus 16-month-old mice. It includes the corresponding fold change (log2FC 3 vs. 16 months) and adjusted *p*-value (*p*-adj). The column “log2FC Neu– vs. Neu+” is based on differential expression analysis comparing transcripts in NeuN− versus NeuN+ nuclei, with a positive value above 4 indicating significant enrichment in NeuN− nuclei. The columns “Conserved” and “Human Homolog” indicate whether a human homolog exists. LncRNAs in bold indicate the presence of a human homolog.

## Data Availability

RNA sequencing data are available via the Gene Expression Omnibus (GEO) database, Accession number: GSE275168.

## Supplementary Materials

The following supporting information can be downloaded at: https://www.mdpi.com/article/10.3390/ncrna11010002/s1.
