# Supplementary material for: LncRNA 3222401L13Rik Is Upregulated in Aging Astrocytes and Regulates Neuronal Support Function Through Interaction with Npas3
Source: Noncoding RNA. 2025 Jan 9;11(1):2. doi: 10.3390/ncrna11010002 (PMC11755665; doi:10.3390/ncrna11010002)
Supplement: Supplementary file 1 [file ncrna-11-00002-s001.zip › ncrna-3356399-Figure S1.pdf]

Supplemental Figure S1

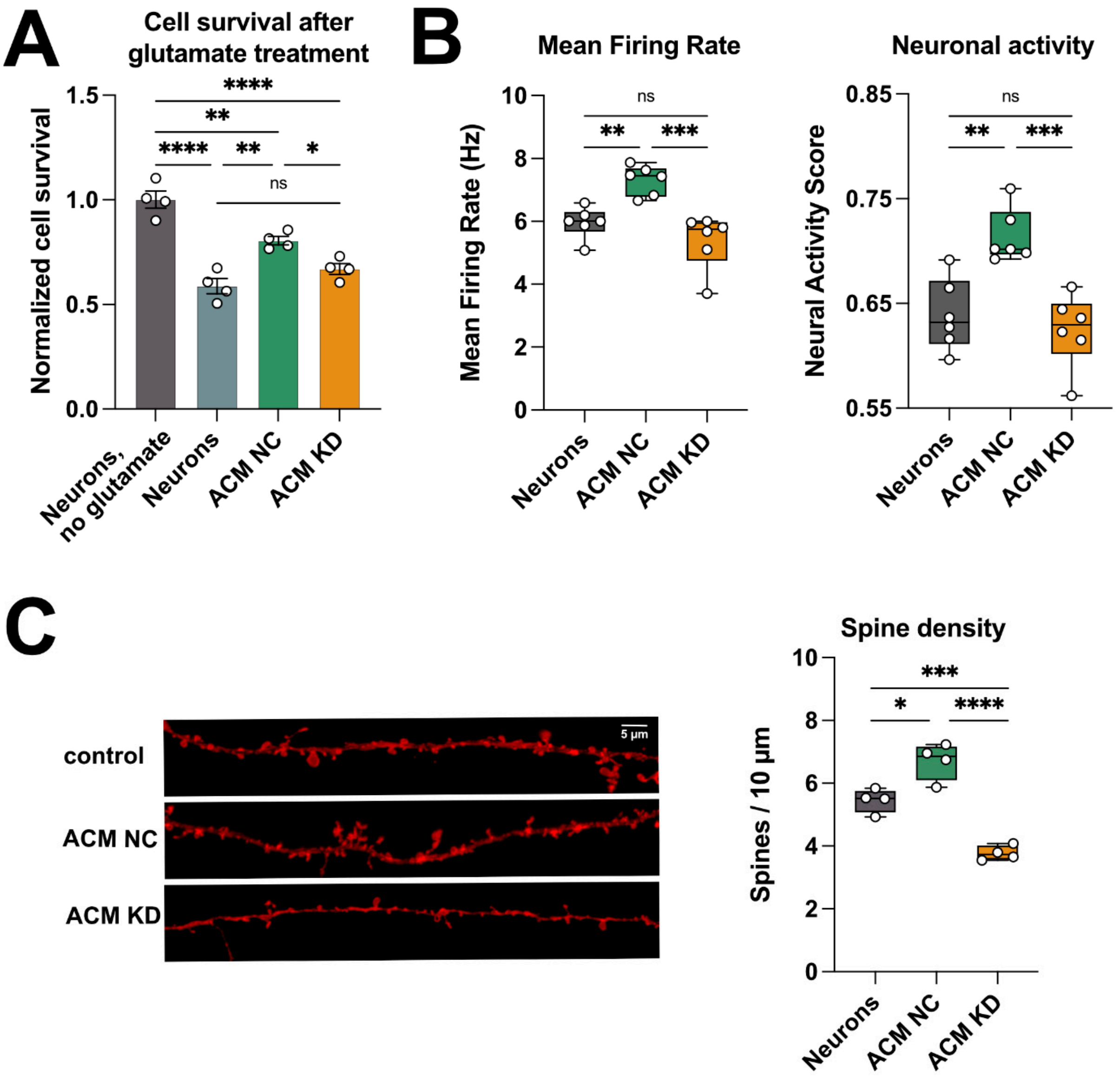

Figure S1. Knock down of *3222401L13Rik* in astrocytes impairs neuronal plasticity. The astrocyte-neuron co-culturing experiments shown in Fig. 4E & F are not feasible in the context of MEA assays, since co-culturing is not possible when cells are grown on MEA plates. Thus, we used a different approach to test electrophysiological properties in neurons upon astrocytic knock down of *3222401L13Rik*. To this end we treated primary astrocytes with GapmeRs targeting *3222401L13Rik* (KD), using or GapmerRs that do not bind any transcript as negative control (NC). Subsequently we harvested the media from these cells (astrocyte conditioned media, ACM) and added this to neurons followed by functional analysis. More specifically in all experiments we added to neuronal cell cultures either control pure NB+ (media control) or ACM from primary astrocytes treated with NC GapmerRs (ACM NC) or primary astrocytes in which *3222401L13Rik* was knocked down (ACM KD).

(A) Survival of neurons after treatment with 100 µM glutamate cultured with control medium (media control), with ACM NC or ACM KD media (One-way ANOVA; *$p < 0.05$, **$p < 0.01$, ****$p < 0.0001$, ns = not significant). **(B)** Left panel: mean firing rate of neurons cultured with normal medium or with ACM from NC or KD astrocytes (One-way ANOVA; **$p < 0.01$, ***$p < 0.001$, ns = not significant). Right panel: Bar plot showing the neural Activity Score obtained from MEA recordings of neurons cultured with normal medium or with ACM from NC or KD astrocytes (One-way ANOVA; **$p < 0.01$, ***$p < 0.001$, ns = not significant). **(C)** Left panel: Representative images of dendrite and spine labeling of neurons cultured with normal medium or with ACM from NC or KD astrocytes. Right panel: Quantification of spines shown in the left panel (One-way ANOVA; *$p < 0.05$, ***$p < 0.001$, ****$p < 0.0001$). Data are depicted as mean ± standard error. NC: negative control, KD: knockdown.
